# Myopia is associated with education: Results from NHANES 1999-2008

**DOI:** 10.1371/journal.pone.0211196

**Published:** 2019-01-29

**Authors:** Stefan Nickels, Susanne Hopf, Norbert Pfeiffer, Alexander K. Schuster

**Affiliations:** 1 Center for Ophthalmic Epidemiology and Healthcare Research, Department of Ophthalmology, University Medical Center of the Johannes Gutenberg-University Mainz, Mainz, Germany; 2 Department of Ophthalmology, University Medical Center of the Johannes Gutenberg-University Mainz, Mainz, Germany; National Yang-Ming University Hospital, TAIWAN

## Abstract

**Purpose:**

Myopia is increasing worldwide and possibly linked to education. In this study, we analyse the association of myopia and education in the U.S. and investigate its age-dependency.

**Methods:**

We conducted a secondary data analysis using the public use files from the cross-sectional study National Health and Nutrition Examination Survey of the period from 1999 to 2008. 19,756 participants aged 20 to 85 years were included with data on education and ophthalmic parameters (distance visual acuity, objective refraction and keratometry). Spherical equivalent, astigmatism, corneal power and corneal astigmatism were evaluated for an association with education using linear regression analysis with adjustment of potential confounders.

**Results:**

Analysis revealed an association between spherical equivalent and educational level in the univariate analysis (P < .001), and in the adjusted model (P < .001). Subjects who attend school to less than 9th grade had a mean spherical equivalent of 0.34 D, subjects with 9-11th grade -0.14 D, subjects that finished high school -0.33 D, subjects with partial college education -0.70 D, subjects that graduated from college or a higher formal education -1.22 D. Subjects that graduated from college or above were -1.47 D more myopic compared to subjects that completed less than 9^th^ grade school in the adjusted analyses. Astigmatism and corneal curvature was not associated with education.

**Conclusions:**

Myopia is associated with higher education in the U.S. Our analysis shows that corneal curvature does not contribute to this association, therefore axial elongation or lens power are likely to contribute to myopia.

## Introduction

Myopia (short-sightedness) is a complex condition leading to blurred vision in distance. To overcome this, dispersing (minus-diopter) lenses or contact lenses are worn to create a sharp image on the retina. Prevalence of myopia has rapidly increased during the last two decades, especially in south-east Asia, but also in the western world [1–4]. Both environmental factors and genetic factors are thought to play a role in the pathogenesis of myopia [5–10]. Several environmental factors are described to be related to prevalence and severity of myopia such as near work and lack of outdoor activity during childhood [11, 12], level of education [13, 14], and residence (urban vs. rural) [15]. Prior research on education and myopia mainly focuses on Asian population [16–19] while less is known about the U.S. population.

Therefore, the purpose of this study was to analyze the association of education with refraction in the U.S.

## Methods

The National Health and Nutrition Examination Survey (NHANES) is a representative survey research program to assess the health and nutritional status of adults in the United States of America (https://www.cdc.gov/nchs/nhanes/index.htm, last accessed 2017-12-11). Since 1999, regular data collection is carried out of approximately 5000 persons from 15 areas, who are examined in two-year periods. Data is raised via questionnaire-based personal interviews at the participant’s home, followed by a visit of a mobile examination center. From 1999 to 2008, NHANES evaluated the level of education and included an ophthalmic examination. Our statistical analyses are based on the NHANES public use files of these survey cycles (https://wwwn.cdc.gov/nchs/nhanes/continuousnhanes/default.aspx, last accessed 2017-12-11). The study design is cross-sectional; NHANES survey protocols were approved by the NCHS Research Ethics Review Board (ERB).

We included all participants of the survey years 1999–2008 aged 20 or older with available information on education and having had an ophthalmic examination. For the variable difference in corneal power between meridians, we excluded negative values (n = 61) and extreme values above 10 diopters (n = 15), as they are highly suspected to be measurement or data entry errors. We excluded all participants with prior refractive surgery (“Have you ever had eye surgery to treat or prevent near-sightedness or myopia?”) (n = 350) or missing information on prior refractive surgery (n = 24) and prior cataract surgery (n = 1,194) or missing information on prior cataract surgery (n = 6). Finally, 19,756 right eyes of study participants were available for analysis.

### Education

Detailed educational attainment information was evaluated asking the question “What is the highest grade or level of school you have completed or the highest degree have received?”. Answer categories were.

1 Less than 9th grade
2 9-11th grade (Includes 12th grade with no diploma)
3 High School grade/GED (General Education Development or Diploma) or equivalent
4 Some College or AA degree
5 College Graduate or above
7 Refused (set to missing)
9 Don't Know (set to missing)
. Missing

AA or Associate of Arts degree is an undergraduate academic degree awarded by colleges usually after completion of a two-year course. GED means General Education Development or Diploma; this certification provides that the test taker has United States or Canadian high-school-level academic skills.

### Ophthalmic data

Study participants took part in an examination of visual function. Objective refraction and keratometry were examined using an autorefractor/keratometer (Nidek ARK-760A, Nidek Co. Ltd., Tokyo, Japan) in non-cycloplegic state. Presenting distance visual acuity was tested in both eyes and, if available, conducted with participants’ own spectacle correction, if available. Objective refraction (sphere, cylinder, axis) and keratometric data was obtained by taking the average of three measurements. Corneal curvature was transformed into corneal power using a keratometric index of r = 1.3375 and corneal power was averaged across the two meridians. Difference in corneal power between meridians was defined as corneal astigmatism and the axis of the steepest meridian was recorded. Myopia was defined as a spherical equivalent ≤ -0.75 diopters [14].

In the analysis of differences in corneal power between meridians participants with negative values and extreme values above 10 diopters were excluded. We furthermore excluded all participants that reported a history of eye surgery including refractive and cataract surgery.

### Demographic data

Age and sex were self-reported, as was ethnicity. Ethnicity was provided in the categories Mexican American, other Hispanic, Non-Hispanic white, Non-Hispanic black, and other.

### Statistical analysis

We included only right eyes in our analysis. Spherical equivalent (SE) was calculated as sphere value plus half the cylindrical power. Visual acuity measures were transformed in the Snellen equivalent to LogMAR [20], the category “20/200+” was set to 1.1 LogMAR. All variables were controlled for outliers.

Following the approach of Thibos [21], who applied Fourier analysis to calculate astigmatism components, namely J_0_ and J_45_, for both refractive and corneal astigmatism as follows:

$$J_{0}=\;-\frac{C}{2}*\cos2\alpha$$

$$J_{45}=\;-\frac{C}{2}*\sin2\alpha$$

α is the cylindrical axis, and C is the cylinder power. J_0_ represents the power vector for the cylinder power of the vertical (90°) and horizontal (180°) meridians. Positive values equal to with-the-rule astigmatism, negative values to against-the-rule astigmatism. J_45_ reflects the cylinder power of the oblique meridians (45° and 135°).

We computed mean and standard deviation for continuous variables showing an approximately normal distribution. Absolute and relative frequencies were computed to describe categorical variables. For continuous variables of interest, we used weighted linear regression models, and for categorical data we conducted weighted logistic regression analyses. As univariate analysis, one model was built for each of the following outcome variable (spherical equivalent, J_0_-vector and J_45_-vector of astigmatism, corneal power, corneal J_0_- and corneal J_45_-vector) with level of education as independent variable. Multivariable regression models included age, sex and NHANES survey cycle. For spherical equivalent, we performed an additional model with adjustment for corneal power. Additional models were adjusted for ethnicity and income as well, as well as for vitamin D levels as a proxy for outdoor activity and sunlight exposure [22, 23]. We performed a sensitivity analysis restricted to participants older than 30 years, since some participants might be still under exposure of education between 20 and 30 years of age. We additionally performed regression models stratified by ethnicity and additionally restricted to US-born participants.

To account for the complex survey design, we followed the NHANES analytical guidelines including combined sample weights for the analyses [24]. Based on the primary sampling units and strata, the variance estimation uses Taylor Series Linearization. In the regression models stratified by ethnicity, we used linear regression models without consideration of the design structure, since numbers in primary sampling units and strata became too small. We used R version 3.3.0 with Rstudio version 1.0.136 and the packages nhanesA (version 0.6.5), ggplot2 (version 2.1.0), survey (version 3.34), knitr (version 1.20), MASS (7.3–45), and tableone (version 0.8.1). P-values should be considered as a continuous measure of evidence and should be cautiously interpreted, given the exploratory character of this analysis. To allow replication of our statistics, we provide the source code on github (https://github.com/snickels/nhanes_edu_se).

## Results

### Sample description

The mean age of our analysis sample (n = 19,756) was 46.6 ± 16.8 years and 10,174 (51.5%) were female. 21.7% where Mexican Americans, 5.9% other Hispanics 47.8% non-Hispanic Whites and 20.7% non-Hispanics Blacks, 3.9% had another ethnicity.

With respect to level of education, 2,457 (12.4%) did reach less than 9th grade, 3,275 (16.6%) 9-11th grade (includes 12th grade with no diploma), 4,805 (24.3%) high school grad/ General Education Development or Diploma (GED) or equivalent, 5,397 (27.3%) some college or AA degree and 3,822 (19.3%) college graduate or above. Subjects with less than 9th grade had a mean spherical equivalent of 0.34 D, subjects with 9-11th grade -0.14 D, subjects that finished high school -0.33 D, subjects with some college education -0.70 D, subjects that graduated from college or a higher formal education -1.22 D.

**Fig 1:**
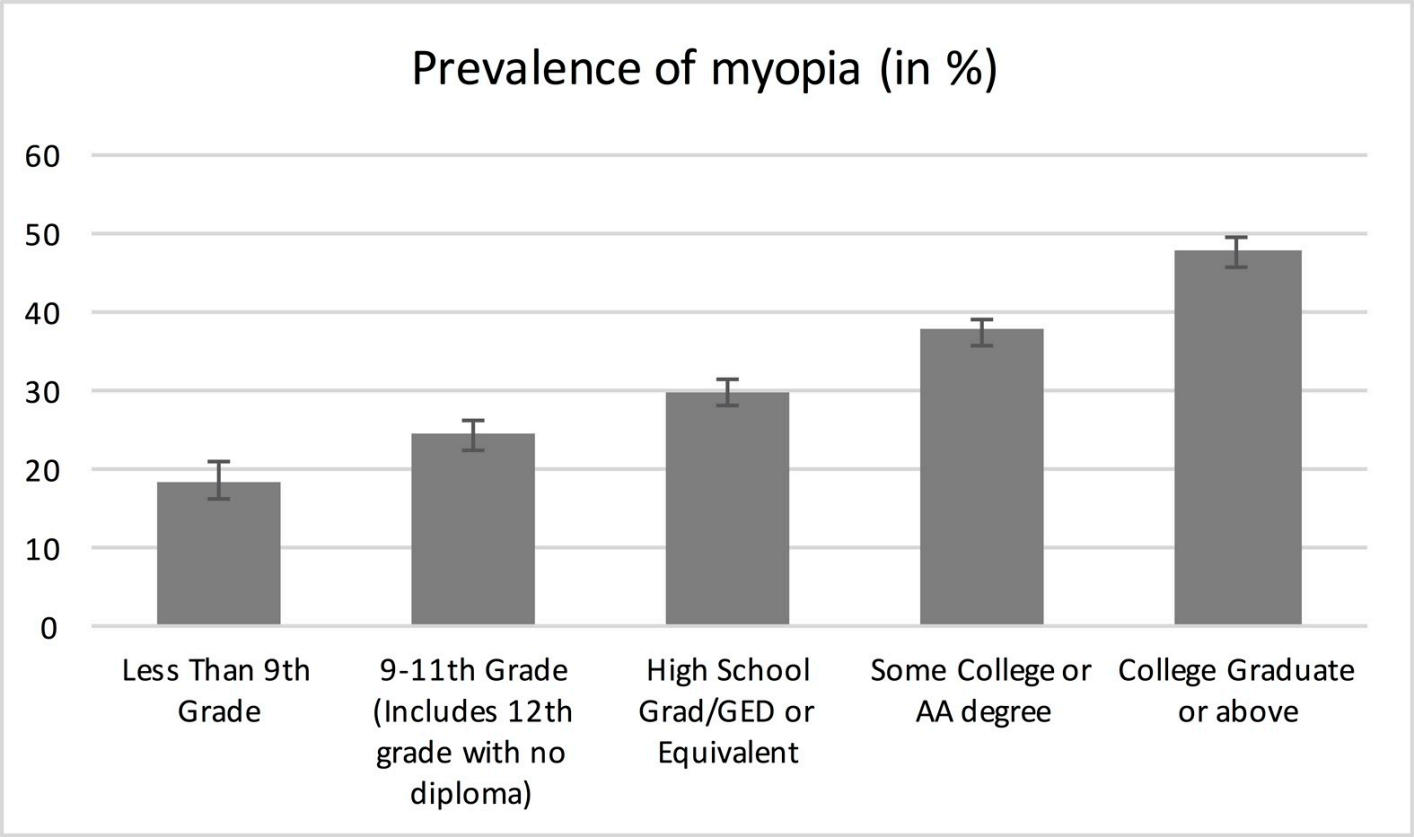
The distribution of myopia (right eyes; ≤ -0.75 D) by level of education in the NHANES 1999–2008.

**Fig 2:**
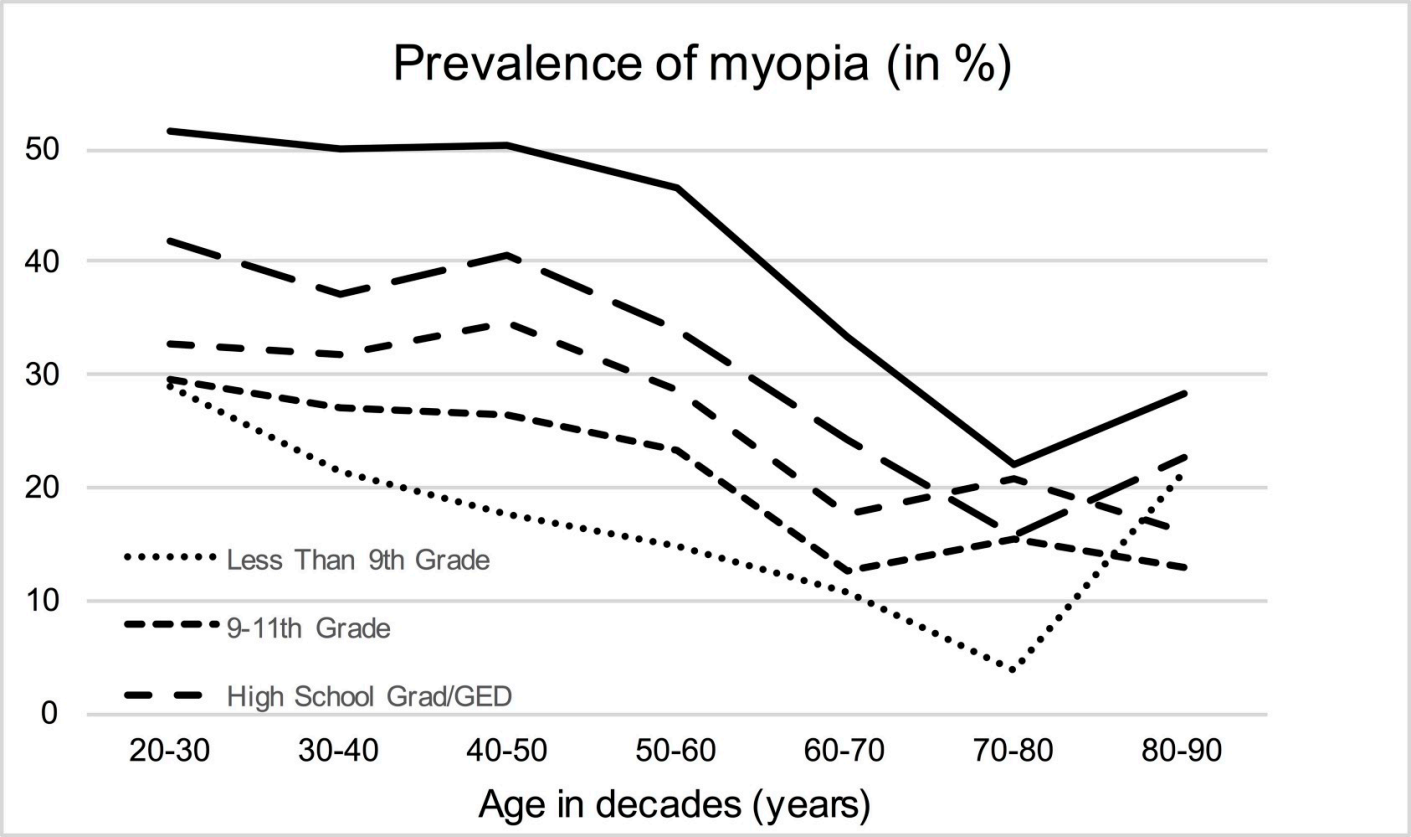
Prevalence of myopia (right eyes; ≤ 0.75D) by level of education and age in the NHANES 1999–2008.

Prevalence of myopia was present in 16.8% of subjects with less than 9th grade, 23.5% with 9-11th grade, 28.6% in subjects that finished high school, 35.4% in subjects with some college education and in 45.0% of those that graduated from college or a higher formal education (Fig 1). These proportions seemed to be relatively constant in the age of 20 to 60 years with only increasing myopia in subjects with education of less than 9^th^ grade (Fig 2), in older study participants myopia was less prevalent. Data of general and ocular parameters are reported in Table 1. In the excluded participants with self-reported refractive surgery, there were more individuals with a higher education compared to the study sample (S1 Table).

**Table 1:** Characteristics of the NHANES 1999–2008 sample with age, sex, education and refraction (right eye) available, by level of education (n = 19,756).

Education	Less Than 9th Grade^a^	9-11th Grade^a^	High School Grad/GED or Equivalent^a^	Some College or AA degree^a^	College Graduate or above^a^
n	2457	3275	4805	5397	3822
Age	53.69 (17.02)	45.38 (17.17)	46.51 (17.30)	44.10 (16.20)	46.67 (15.32)
Female sex	1159 (47.2)	1668 (50.9)	2439 (50.8)	2965 (54.9)	1947 (50.9)
Family income to poverty index^a^	1.47 (1.02)	1.82 (1.33)	2.47 (1.49)	2.90 (1.56)	3.93 (1.39)
Ethnicity^a^:					
Mexican American	1524 (62.0)	915 (27.9)	816 (17.0)	770 (14.3)	265 (6.9)
Other Hispanic	241 (9.8)	244 (7.5)	226 (4.7)	298 (5.5)	159 (4.2)
Non-Hispanic White	403 (16.4)	1001 (30.6)	2625 (54.6)	2865 (53.1)	2552 (66.8)
Non-Hispanic Black	219 (8.9)	1029 (31.4)	982 (20.4)	1253 (23.2)	598 (15.6)
Other	70 (2.8)	86 (2.6)	156 (3.2)	211 (3.9)	248 (6.5)
Ocular characteristics					
Sphere [D]	-0.12 (1.74)	-0.58 (2.03)	-0.76 (2.20)	-1.11 (2.42)	-1.62 (2.68)
Cylinder [D]	0.92 (0.83)	0.87 (0.86)	0.86 (0.79)	0.81 (0.78)	0.80 (0.67)
Spherical Equivalent [D]	0.34 (1.66)	-0.14 (1.91)	-0.33 (2.11)	-0.70 (2.32)	-1.22 (2.62)
Spherical Equivalent < = -0.75D (%)	413 (16.8)	771 (23.5)	1372 (28.6)	1912 (35.4)	1721 (45.0)
Spherical Equivalent (%)					
>-0.75 D	2044 (83.2)	2504 (76.5)	3433 (71.4)	3485 (64.6)	2101 (55.0)
-0.75/-3 D	357 (14.5)	578 (17.6)	956 (19.9)	1224 (22.7)	912 (23.9)
-3/-6 D	47 (1.9)	144 (4.4)	324 (6.7)	491 (9.1)	583 (15.3)
< = -6 D	9 (0.4)	49 (1.5)	92 (1.9)	197 (3.7)	226 (5.9)
Distance glasses (%)	830 (33.8)	1161 (35.5)	2208 (46.0)	2682 (49.7)	2333 (61.0)
Refractive astigmatism >0.5 D (%)	1340 (54.5)	1577 (48.2)	2474 (51.5)	2560 (47.4)	1897 (49.6)
With-the-rule astigmatism	628 (25.6)	601 (18.4)	1033 (21.5)	1003 (18.6)	775 (20.3)
Against-the-rule astigmatism	225 (9.2)	405 (12.4)	583 (12.1)	637 (11.8)	450 (11.8)
Oblique astigmatism	751 (30.6)	960 (29.3)	1394 (29.0)	1574 (29.2)	1130 (29.6)
Refractive Astigmatism J_0_	-0.11 (0.53)	-0.02 (0.54)	-0.06 (0.51)	-0.04 (0.50)	-0.06 (0.45)
Refractive Astigmatism J_45_	0.05 (0.30)	0.03 (0.29)	0.00 (0.28)	0.00 (0.27)	-0.01 (0.25)
Keratometric power [D]	43.63 (1.55)	43.62 (1.55)	43.67 (1.55)	43.71 (1.57)	43.68 (1.55)
Corneal astigmatism [D]	0.98 (0.81)	1.03 (0.84)	0.95 (0.75)	0.95 (0.75)	0.88 (0.61)
Corneal Astigmatism >0.5 D (%)	1545 (63.2)	2125 (65.2)	3021 (63.0)	3430 (63.7)	2320 (60.7)
With-the-rule corneal astigmatism	265 (10.8)	161 (4.9)	290 (6.0)	272 (5.1)	239 (6.3)
Against-the-rule corneal astigmatism	709 (29.0)	1228 (37.7)	1790 (37.3)	2105 (39.1)	1329 (34.8)
Oblique corneal astigmatism	828 (33.9)	1067 (32.7)	1438 (30.0)	1626 (30.2)	1182 (31.0)
Corneal Astigmatism J_0_	0.18 (0.62)	0.30 (0.53)	0.26 (0.52)	0.28 (0.50)	0.23 (0.44)
Corneal Astigmatism J_45_	0.08 (0.40)	0.07 (0.36)	0.03 (0.36)	0.02 (0.32)	0.01 (0.31)
Visual acuity [logMAR]	0.20 (0.25)	0.15 (0.22)	0.13 (0.20)	0.11 (0.19)	0.09 (0.16)

^a^self-reported;

AA: Associate of Arts degree, undergraduate academic degree awarded by colleges usually after completion of a two-year course; GED: General Education Development or Diploma, certification that provides that the test taker has United States or Canadian high-school-level academic skills.

When stratified by ethnicity, non-Hispanic Whites and participants of other ethnicity had the highest percentage of both college graduate and myopia (S2 Table).

### Association analysis

Linear regression analysis revealed an association between myopic spherical equivalent and higher level of education in the univariate analysis (Table 2), and in the model adjusted for age, sex and NHANES examination cycle (Table 2). Our analysis showed a monotonic increase of myopia with higher level of education. The association was still present when additionally adjusting for ethnicity and corneal power, and also when restricting the analysis to participants older than 30 years (S3 and S5 Tables).

**Table 2:** The association of spherical equivalent with education in separate models in the NHANES 1999–2008.

	Crude analysis (n = 19,756)		Adjusted model^a^ (n = 19,756)		Adjusted model^b^ (n = 19,704)	
Education	Estimate in diopter [95% CI]	P value	Estimate in diopter [95% CI]	P value	Estimate in diopter [95% CI]	P value
Less Than 9th Grade	Reference	-	Reference	-	Reference	-
9-11th Grade	-0.45 [-0.60; -0.30]	1.02e-07	-0.23 [-0.36; -0.10]	0.001	-0.25 [-0.39; -0.11]	< 0.001
High School Grad/GED or Equivalent	-0.68 [-0.83; -0.52]	1.87e-12	-0.49 [-0.63; -0.35]	2.22e-09	-0.48 [-0.62; -0.34]	9.44e-09
Some College or AA degree	-1.07 [-1.22; -0.92]	< 2e-16	-0.80 [-0.93; -0.66]	< 2e-16	-0.79 [-0.92; -0.65]	< 2e-16
College Graduate or above	-1.65 [-1.83; -1.48]	< 2e-16	-1.47 [-1.63; -1.30]	< 2e-16	-1.46 [-1.62; -1.29]	< 2e-16

All models calculated with consideration of the study sample structure;

^a^results from the multivariable linear regression models adjusted for age, sex, survey cycle;

^b^additionally adjusted for corneal power;

AA: Associate of Arts degree, undergraduate academic degree awarded by colleges usually after completion of a two-year course; GED: General Education Development or Diploma, certification that provides that the test taker has United States or Canadian high-school-level academic skills; CI: confidence interval

Logistic regression analysis showed that myopia ≤ -0.75D is associated with higher level of education. The higher the level of education, the more likely the study participants had a myopic refractive error (Table 3). Adjustment for potential confounders did not alter this finding (Tables 3 and S4), as well as restricting to participants older than 30 years (S6 Table).

**Table 3:** The association of myopia (≤ -0.75 D) with education in separate models in the NHANES 1999–2008.

	Crude analysis (n = 19,756)		Adjusted model^a^ (n = 19,756)		Adjusted model^b^ (n = 19,704)	
Education	Odds ratio [95% CI]	P value	Odds ratio [95% CI]	P value	Odds ratio [95% CI]	P value
Less Than 9th Grade	Reference	-	Reference	-	Reference	-
9-11th Grade	1.42 [1.15; 1.74]	0.002	1.27 [1.04; 1.57]	0.02	1.30 [1.05; 1.61]	0.02
High School Grad/GED or Equivalent	1.87 [1.57; 2.23]	1.64e-09	1.71 [1.43; 2.03]	9.55e-08	1.71 [1.42; 2.04]	2.39e-07
Some College or AA degree	2.66 [2.22; 3.19]	3.96e-16	2.32 [1.94; 2.78]	3.56e-13	2.34 [1.93; 2.83]	1.59e-12
College Graduate or above	4.05 [3.37; 4.86]	< 2e-16	3.74 [3.12; 4.48]	< 2e-16	3.81 [3.15; 4.60]	< 2e-16

All models calculated with consideration of the study sample structure;

^a^results from the multivariable logistic regression models adjusted for age, sex, survey cycle;

^b^additionally adjusted for corneal power;

AA: Associate of Arts degree, undergraduate academic degree awarded by colleges usually after completion of a two-year course; GED: General Education Development or Diploma, certification that provides that the test taker has United States or Canadian high-school-level academic skills; CI: confidence interval.

Additional adjustment for vitamin D levels as a proxy for outdoor activity had only minor influence on estimates (S3 and S4 Tables). In the analysis stratified by ethnicity, the effect estimates were similar across most ethnicities except for the Non-Hispanic Black and the other ethnicity group, in which the estimates for the association of education with myopia were higher (S7 and S8 Tables). We additionally restricted to US-born participants, assuming a more uniform exposure to the US education. The effect for the Non-Hispanic black and the “other” group were higher than for other ethnicities (Mexican American, Non-Hispanic white) but the precision of the estimates was low due to the small sample size (S9 and S10 Tables).

Neither an association was present between corneal power with level of education (Table 4), nor for refractive astigmatism. For corneal astigmatism, only subjects graduated from college or a higher formal education had a slightly lower astigmatism (Table 5). Oblique corneal and refractive astigmatism (J_45_) was less present in subjects with higher educational level (S11 and S12 Tables).

**Table 4:** Association analysis of corneal power and corneal astigmatism with level of education in the NHANES 1999–2008.

Corneal power				
	Crude analysis (n = 19,704)		Adjusted model^a^ (n = 19,704)	
Education	B [95% CI]	P value	B [95% CI]	P value
Less Than 9th Grade	Reference	-	Reference	-
9-11th Grade	-0.03 [-0.13; 0.08]	0.65	-0.06 [-0.16; 0.04]	0.22
High School Grad/GED or Equivalent	0.07 [-0.04; 0.18]	0.21	0.04 [-0.06; 0.14]	0.43
Some College or AA degree	0.10 [0.00; 0.20]	0.05	0.04 [-0.05; 0.14]	0.39
College Graduate or above	0.07 [-0.03; 0.16]	0.18	0.03 [-0.06; 0.13]	0.49

Results from the multivariable linear regression models adjusted for age, sex, survey cycle, and with consideration of the study sample structure.

All models calculated with consideration of the study sample structure;

^a^results from the multivariable linear regression models adjusted for age, sex, survey cycle;

AA: Associate of Arts degree, undergraduate academic degree awarded by colleges usually after completion of a two-year course; GED: General Education Development or Diploma, certification that provides that the test taker has United States or Canadian high-school-level academic skills.

**Table 5:** The association of refractive and corneal astigmatism with education in separate models in the NHANES 1999–2008.

	refractive astigmatism (> 0.5 D cylinder power)				corneal astigmatism (> 0.5 D cylinder power)			
	Crude analysis (n = 19,756)		Adjusted model^a^ (n = 19,756)		Crude analysis (n = 19,704)		Adjusted model^a^ (n = 19,704)	
Education	Odds ratio [95% CI]	P value	Odds ratio [95% CI]	P value	Odds ratio [95% CI]	P value	Odds ratio [95% CI]	P value
Less Than 9th Grade	Reference	-	Reference	-	Reference	-	Reference	-
9-11th Grade	-0.19 [-0.29;-0.09]	0.0004	0.00 [-0.11; 0.12]	0.98	0.02 [-0.15; 0.20]	0.79	-0.05 [-0.23; 0.13]	0.57
High School Grad/GED or Equivalent	-0.03 [-0.14; 0.09]	0.65	0.13 [0.02; 0.25]	0.03	-0.07 [-0.22; 0.08]	0.34	-0.12 [-0.27; 0.03]	0.11
Some College or AA degree	-0.16 [-0.28;-0.04]	0.01	0.07 [-0.06; 0.20]	0.27	-0.05 [-0.20; 0.11]	0.56	-0.12 [-0.27; 0.03]	0.12
College Graduate or above	-0.07 [-0.20; 0.05]	0.26	0.08 [-0.04; 0.21]	0.18	-0.17 [-0.33; -0.02]	0.03	-0.22 [-0.38; -0.07]	0.01

All models calculated with consideration of the study sample structure;

^a^results from the multivariable logistic regression models adjusted for age, sex, survey cycle;

AA: Associate of Arts degree, undergraduate academic degree awarded by colleges usually after completion of a two-year course; GED: General Education Development or Diploma, certification that provides that the test taker has United States or Canadian high-school-level academic skills.

## Discussion

Our analysis provides data on the refractive error and corneal properties, namely corneal power and corneal astigmatism in adults aged 20 to 85 years with respect to their level of education in a population based setting in the U.S. It indicates that a higher level of education is linked to myopic refractive error, while astigmatism and corneal power are not associated.

Despite differences in the educational system, similar results with respect to myopic refractive error have been reported in other population-based and cohort studies [25–37]. Hyperopia in adults has been proven to be associated with lower educational qualifications in the U.K. and the strength of the association increases with the level of refractive error in both directions [34].

Mirshahi et al. reported that individuals who graduated after 13 years of school were more myopic (median: -0.5D) than those graduating after 10 years (-0.2D), after 9 years (+0.3D) or never finishing secondary school (+0.2D) [13]. Similar relationships were found in our data analysis, showing a stepwise increase of myopia level and prevalence with higher education in the U.S.: i.e. persons graduating from college were -1.46D more myopic than persons finish school education with less than 9^th^ grade. Similar, prevalence of myopia (≤ -0.75D) was 45% in persons graduating from college was more prevalent than in subjects with some college education (35%), in subjects that finished high school (28%), in those with 9-11^th^ grade education (24%) and those with less than 9^th^ grade (17%). The U.S. prevalence data of myopia are in line with the E3 data from Williams et al. reporting higher prevalence of myopia in individuals with higher education (36.6%) compared to those who completed secondary (29.1%) or primary education (25.4%) [14]. The myopic shift seems to persist when attending university: Kinge et al. reported that university students underwent a -0.5D shift in refractive error over a 3-years observational period, mainly due to vitreous chamber elongation [38].

This impact of education on myopic refractive error also seems to be affected by year of birth. Williams et al. reported that the association of education with myopia is dependent on age (respective decade of birth): while in the 1920s only 25% of subjects with higher education (leaving school at age of ≥ 20 years) were myopic, in the 1950s and 1960s about 40% of subjects with this educational level were myopic [14]. The former National Health and Nutrition Examination Survey from 1971 to 1972 revealed a prevalence of 25% myopic individuals aged 12 to 54 years and prevalence rates increased with educational level [39]. In contrast, Liang et al. reported for rural Chinese that higher level of education was linked to myopia in ≥50 years of age, but not in younger subjects (30 to 49 years) [40]. In these younger subjects in the Handan region, hours of reading were associated with myopia. In our study however, myopia prevalence in the different educational levels were relatively constant in the age of 20 to 60 years, only subjects with education of less than 9^th^ grade showed a clear increase. In older study participants (60 to 90 years), myopia was less prevalent. Other studies from Asia reported a larger effect of education on myopia than we observed. In an Indian population, higher education was associated with a nearly two-fold increased risk for high myopia. A South Korean study [41] reported a two-fold increased risk for myopia and a study from Singapore a four-fold increased risk for myopia associated with a University degree [18]. A recent meta-analysis provided evidence for stronger effects of high education in individuals of Asian ancestry than in European ancestry [8]. We were not able to sufficiently explore the subsample of Asian descent in our analysis. The category “Non-Hispanic Asian” was not available until NHANES survey cycle 2011–12: In our analysis, participants of Asian descent are summarized together with other ethnicities in the “other” category, and this might have diluted the effect.

Another explanation might be differences between Asian and US-American education systems. We have therefore performed a sensitivity analysis with U.S. born study participant receiving most likely their school education in the U.S. In addition, different patterns of out-of-school activities [42] might lead to differences between Asian and US-American education systems with respect to myopia.

Rather the environmental factors of education play a role than cognitive function of the myopic subjects. A recent population-based analysis by Mirshahi et al. reported that myopia itself is independent of cognitive function after adjusting for level of education [43]. In contrast, IQ test results and years in education were related to myopia in Danish conscripts [44]. The SCORM (Singapore Cohort study Of Risk factors for Myopia) revealed nonverbal IQ, reading and school grades as risk factors for myopia and high/higher myopia in children [45, 46].

The combination of good educational outcome and extra classes after school is related to higher prevalence of myopia as shown in an analysis of the PISA2009 data [47]. The impact of education on refractive error has been proven in countries with > 90% myopes such as Korea [48].

### Astigmatism

In our study, cylindrical power of refraction was not associated with level of education. While astigmatism with respect to with/against the rule (J_0_-vector) was not associated, oblique astigmatism (J_45_-vector) decreased with increasing education. Literature shows different findings for the relationship between astigmatism and education. Hashemi et al. reported an inverse association of educational level and astigmatism in rural areas of Iran [49], Rim et al. found decreased prevalence of astigmatism in highly educated Korean subjects [26], while other studies did not find an association of astigmatism with level of education [28, 32].

### Corneal power

Similar to literature, we did not find a relationship between corneal power and level of education. Jonas et al. reported that corneal curvature in adults is not associated with level of education in the Beijing Eye Study and the Central India Eye and Medical Study [50]. Comparable findings were reported by Lim et al. [51] or Lee et al. [52]

### Strengths and limitations

The strength of the NHANES study lies in the large sample size and the standardized population-based study design. The adjustment for different covariates allows to precise estimates. A limitation in this study is the use of non-cycloplegic refraction date. The analysis done may have underestimated some hyperopes, which is why we performed a sensitivity analysis including only myopic study participants and did find comparable associations as for the total study cohort.

We excluded all participants with self-reported refractive surgery for myopia, as we were not able to identify the refractive error before treatment. These participants showed a higher education compared to the study group and by excluding them, a bias might have been introduced.

In addition, we were not able to include information on presence of cataract nor genetic factors as potential risk factors. Nevertheless, Mirshahi et al. showed that environmental factors including education play the major role while genes have less impact on myopia [13], accounting for less than 5% of the variance of refractive error [53]. In the past, two types of high myopia were suspected based on dependency on genetic background and age of onset that also differed in associations with education as well: in the non-adult study groups, myopia increased with higher degree of education, while not in the adult participants of the Beijing Eye Study and the Central India Eye and Medical Study [54]. This finding may be driven by the underlying educational status of the population that is different between industrialised and newly industrialised countries.

## Conclusion

In conclusion, myopia is associated with higher school and college education in the U.S. Our analysis shows that corneal curvature does not cause this association, therefore other parameters may be regarded as underlying factors for myopia in higher educated persons.

## Supporting information

S1 Table Comparison of the NHANES 1999–2008 sample without and with self-reported refractive surgery for near sightedness (excluded from analysis). (PDF)

S2 Table Characteristics of the NHANES 1999–2008 sample with age, sex, education and refraction (right eye) available, by ethnicity (n = 19,756). (PDF)

S3 Table The association of spherical equivalent with education in separate models in the NHANES 1999–2008, with additional adjustment. (PDF)

S4 Table The association of myopia (≤ -0.75 D) with education in separate models in the NHANES 1999–2008, with additional adjustment. (PDF)

S5 Table Sensitivity analysis: The association of spherical equivalent with education in separate models in the NHANES 1999–2008, restricted to participants aged 30 years and older. (PDF)

S6 Table Sensitivity analysis: The association of myopia (≤ -0.75 D) with education in separate models in the NHANES 1999–2008, restricted to participants aged 30 years and older. (PDF)

S7 Table The association of spherical equivalent with education in different ethnicities in the NHANES 1999–2008. (PDF)

S8 Table The association of myopia (≤ -0.75 D) with education in different ethnicities in the NHANES 1999–2008. (PDF)

S9 Table The association of spherical equivalent with education in different ethnicities in the NHANES 1999–2008, restricted to US-born participants. (PDF)

S10 Table The association of myopia (≤ -0.75 D) with education in different ethnicities in the NHANES 1999–2008, restricted to US-born participants. (PDF)

S11 Table Association analysis of refractive astigmatism with level of education in the NHANES 1999–2008. (PDF)

S12 Table Association analysis of corneal astigmatism (J0 and J45 vector) with level of education in the NHANES 1999–2008. (PDF)

## References

[1] Buch H, Vinding T, La Cour M, Appleyard M, Jensen GB, Nielsen NV. Prevalence and causes of visual impairment and blindness among 9980 Scandinavian adults: the Copenhagen City Eye Study. Ophthalmology. 2004;111(1):53–61. 14711714 10.1016/j.ophtha.2003.05.010

[2] Iwase A, Araie M, Tomidokoro A, Yamamoto T, Shimizu H, Kitazawa Y. Prevalence and causes of low vision and blindness in a Japanese adult population: the Tajimi Study. Ophthalmology. 2006;113(8):1354–62. 10.1016/j.ophtha.2006.04.022 16877074

[3] Vitale S, Sperduto RD, Ferris FL 3rd. Increased prevalence of myopia in the United States between 1971–1972 and 1999–2004. Archives of ophthalmology (Chicago, Ill: 1960). 2009;127(12):1632–9. 10.1001/archophthalmol.2009.303 20008719

[4] Foster PJ, Jiang Y. Epidemiology of myopia. Eye (London, England). 2014;28(2):202–8. 10.1038/eye.2013.280 PMC3930282 24406412

[5] Morgan IG, Ohno-Matsui K, Saw SM. Myopia. Lancet (London, England). 2012;379(9827):1739–48. 10.1016/S0140-6736(12)60272-4 22559900

[6] Verhoeven VJ, Hysi PG, Wojciechowski R, Fan Q, Guggenheim JA, Hohn R, et al Genome-wide meta-analyses of multiancestry cohorts identify multiple new susceptibility loci for refractive error and myopia. Nat Genet. 2013;45(3):314–8. 10.1038/ng.2554 23396134 PMC3740568

[7] Morgan IG, French AN, Ashby RS, Guo X, Ding X, He M, et al The epidemics of myopia: Aetiology and prevention. Progress in retinal and eye research. 2018;62:134–49. 10.1016/j.preteyeres.2017.09.004 28951126

[8] Fan Q, Verhoeven VJ, Wojciechowski R, Barathi VA, Hysi PG, Guggenheim JA, et al Meta-analysis of gene-environment-wide association scans accounting for education level identifies additional loci for refractive error. Nature communications. 2016;7:11008 10.1038/ncomms11008 27020472 PMC4820539

[9] Guggenheim JA, St Pourcain B, McMahon G, Timpson NJ, Evans DM, Williams C. Assumption-free estimation of the genetic contribution to refractive error across childhood. Molecular vision. 2015;21:621–32. 26019481 PMC4445077

[10] Hwang HS, Park GH, Heo JW, Kim MK, Baek SH, Cho BJ. Estimating heritability of refractive error in Koreans: the Korea National Health and Nutrition Examination Survey. Acta ophthalmologica. 2018. 10.1111/aos.13915 30207075

[11] Guo Y, Liu LJ, Xu L, Lv YY, Tang P, Feng Y, et al Outdoor activity and myopia among primary students in rural and urban regions of Beijing. Ophthalmology. 2013;120(2):277–83. 10.1016/j.ophtha.2012.07.086 23098368

[12] Li SM, Li SY, Kang MT, Zhou Y, Liu LR, Li H, et al Near Work Related Parameters and Myopia in Chinese Children: the Anyang Childhood Eye Study. PloS one. 2015;10(8):e0134514 10.1371/journal.pone.0134514 26244865 PMC4526691

[13] Mirshahi A, Ponto KA, Hoehn R, Zwiener I, Zeller T, Lackner K, et al Myopia and level of education: results from the Gutenberg Health Study. Ophthalmology. 2014;121(10):2047–52. 10.1016/j.ophtha.2014.04.017 24947658

[14] Williams KM, Bertelsen G, Cumberland P, Wolfram C, Verhoeven VJ, Anastasopoulos E, et al Increasing Prevalence of Myopia in Europe and the Impact of Education. Ophthalmology. 2015;122(7):1489–97. 10.1016/j.ophtha.2015.03.018 25983215 PMC4504030

[15] Xu L, You QS, Jonas JB. Refractive error, ocular and general parameters and ophthalmic diseases. The Beijing Eye Study. Graefe's archive for clinical and experimental ophthalmology = Albrecht von Graefes Archiv fur klinische und experimentelle Ophthalmologie. 2010;248(5):721–9. 10.1007/s00417-009-1233-0 19937051

[16] Au Eong KG, Tay TH, Lim MK. Education and myopia in 110,236 young Singaporean males. Singapore medical journal. 1993;34(6):489–92. 8153707

[17] Fan Q, Wojciechowski R, Kamran Ikram M, Cheng CY, Chen P, Zhou X, et al Education influences the association between genetic variants and refractive error: a meta-analysis of five Singapore studies. Human molecular genetics. 2014;23(2):546–54. 10.1093/hmg/ddt431 24014484 PMC3869359

[18] Pan CW, Zheng YF, Anuar AR, Chew M, Gazzard G, Aung T, et al Prevalence of refractive errors in a multiethnic Asian population: the Singapore epidemiology of eye disease study. Investigative ophthalmology & visual science. 2013;54(4):2590–8. 23513059 10.1167/iovs.13-11725

[19] Wu HM, Seet B, Yap EP, Saw SM, Lim TH, Chia KS. Does education explain ethnic differences in myopia prevalence? A population-based study of young adult males in Singapore. Optometry and vision science: official publication of the American Academy of Optometry. 2001;78(4):234–9. 11349931 10.1097/00006324-200104000-00012

[20] Holladay JT. Proper method for calculating average visual acuity. Journal of refractive surgery (Thorofare, NJ: 1995). 1997;13(4):388–91. 10.3928/1081-597X-19970701-16 9268940

[21] Thibos LN, Wheeler W, Horner D. Power vectors: an application of Fourier analysis to the description and statistical analysis of refractive error. Optometry and vision science: official publication of the American Academy of Optometry. 1997;74(6):367–75. 9255814 10.1097/00006324-199706000-00019

[22] Scragg R, Camargo CA Jr. Frequency of leisure-time physical activity and serum 25-hydroxyvitamin D levels in the US population: results from the Third National Health and Nutrition Examination Survey. American journal of epidemiology. 2008;168(6):577–86; discussion 87–91. 10.1093/aje/kwn163 18579538 PMC2727193

[23] Pan CW, Qian DJ, Saw SM. Time outdoors, blood vitamin D status and myopia: a review. Photochemical & photobiological sciences: Official journal of the European Photochemistry Association and the European Society for Photobiology. 2017;16(3):426–32. 10.1039/c6pp00292g 27921098

[24] Johnson CL, Paulose-Ram R, Ogden CL, Carroll MD, Kruszon-Moran D, Dohrmann SM, et al National health and nutrition examination survey: analytic guidelines, 1999–2010. Vital and health statistics Series 2, Data evaluation and methods research. 2013;(161):1–24. 25090154

[25] Varma R, Torres M, McKean-Cowdin R, Rong F, Hsu C, Jiang X. Prevalence and Risk Factors for Refractive Error in Adult Chinese Americans: The Chinese American Eye Study. American journal of ophthalmology. 2017;175:201–12. 10.1016/j.ajo.2016.10.002 27769895 PMC5337169

[26] Rim TH, Kim SH, Lim KH, Choi M, Kim HY, Baek SH. Refractive Errors in Koreans: The Korea National Health and Nutrition Examination Survey 2008–2012. Korean journal of ophthalmology: KJO. 2016;30(3):214–24. 10.3341/kjo.2016.30.3.214 27247521 PMC4878982

[27] Pan CW, Klein BE, Cotch MF, Shrager S, Klein R, Folsom A, et al Racial variations in the prevalence of refractive errors in the United States: the multi-ethnic study of atherosclerosis. American journal of ophthalmology. 2013;155(6):1129–38.e1. 10.1016/j.ajo.2013.01.009 23453694 PMC3759975

[28] Tan CS, Chan YH, Wong TY, Gazzard G, Niti M, Ng TP, et al Prevalence and risk factors for refractive errors and ocular biometry parameters in an elderly Asian population: the Singapore Longitudinal Aging Study (SLAS). Eye (London, England). 2011;25(10):1294–301. 10.1038/eye.2011.144 PMC3194304 21720418

[29] Saw SM, Chan YH, Wong WL, Shankar A, Sandar M, Aung T, et al Prevalence and risk factors for refractive errors in the Singapore Malay Eye Survey. Ophthalmology. 2008;115(10):1713–9. 10.1016/j.ophtha.2008.03.016 18486221

[30] Cheng CY, Hsu WM, Liu JH, Tsai SY, Chou P. Refractive errors in an elderly Chinese population in Taiwan: the Shihpai Eye Study. Investigative ophthalmology & visual science. 2003;44(11):4630–8. 14578378 10.1167/iovs.03-0169

[31] Dandona R, Dandona L, Naduvilath TJ, Srinivas M, McCarty CA, Rao GN. Refractive errors in an urban population in Southern India: the Andhra Pradesh Eye Disease Study. Investigative ophthalmology & visual science. 1999;40(12):2810–8. 10549640

[32] Katz J, Tielsch JM, Sommer A. Prevalence and risk factors for refractive errors in an adult inner city population. Investigative ophthalmology & visual science. 1997;38(2):334–40. 9040465

[33] Attebo K, Ivers RQ, Mitchell P. Refractive errors in an older population: the Blue Mountains Eye Study. Ophthalmology. 1999;106(6):1066–72. 10.1016/S0161-6420(99)90251-8 10366072

[34] Cumberland PM, Bao Y, Hysi PG, Foster PJ, Hammond CJ, Rahi JS. Frequency and Distribution of Refractive Error in Adult Life: Methodology and Findings of the UK Biobank Study. PloS one. 2015;10(10):e0139780 10.1371/journal.pone.0139780 26430771 PMC4591976

[35] Wang Q, Klein BE, Klein R, Moss SE. Refractive status in the Beaver Dam Eye Study. Investigative ophthalmology & visual science. 1994;35(13):4344–7. 8002254

[36] Richler A, Bear JC. Refraction, nearwork and education. A population study in Newfoundland. Acta Ophthalmol (Copenh). 1980;58(3):468–78. 7415832 10.1111/j.1755-3768.1980.tb05748.x

[37] Wensor M, McCarty CA, Taylor HR. Prevalence and risk factors of myopia in Victoria, Australia. Archives of ophthalmology (Chicago, Ill: 1960). 1999;117(5):658–63. 10.1001/archopht.117.5.658 10326965

[38] Kinge B, Midelfart A, Jacobsen G, Rystad J. Biometric changes in the eyes of Norwegian university students—a three-year longitudinal study. Acta ophthalmologica Scandinavica. 1999;77(6):648–52. 10634556 10.1034/j.1600-0420.1999.770608.x

[39] Sperduto RD, Seigel D, Roberts J, Rowland M. Prevalence of myopia in the United States. Archives of ophthalmology (Chicago, Ill: 1960). 1983;101(3):405–7. 10.1001/archopht.1983.01040010405011 6830491

[40] Liang YB, Wong TY, Sun LP, Tao QS, Wang JJ, Yang XH, et al Refractive errors in a rural Chinese adult population the Handan eye study. Ophthalmology. 2009;116(11):2119–27. 10.1016/j.ophtha.2009.04.040 19744728

[41] Kim EC, Morgan IG, Kakizaki H, Kang S, Jee D. Prevalence and risk factors for refractive errors: Korean National Health and Nutrition Examination Survey 2008–2011. PloS one. 2013;8(11):e80361 10.1371/journal.pone.0080361 24224049 PMC3818255

[42] Won SJ, Han S. Out-of-School Activities and Achievement Among Middle School Students in the U.S. and South Korea. Journal of Advanced Academics. 2010;21(4):628–61.

[43] Mirshahi A, Ponto KA, Laubert-Reh D, Rahm B, Lackner KJ, Binder H, et al Myopia and Cognitive Performance: Results From the Gutenberg Health Study. Investigative ophthalmology & visual science. 2016;57(13):5230–6. 27701634 10.1167/iovs.16-19507

[44] Jacobsen N, Jensen H, Goldschmidt E. Prevalence of myopia in Danish conscripts. Acta ophthalmologica Scandinavica. 2007;85(2):165–70. 10.1111/j.1600-0420.2006.00789.x 17305729

[45] Saw SM, Cheng A, Fong A, Gazzard G, Tan DT, Morgan I. School grades and myopia. Ophthalmic & physiological optics: the journal of the British College of Ophthalmic Opticians (Optometrists). 2007;27(2):126–9. 17324201 10.1111/j.1475-1313.2006.00455.x

[46] Saw SM, Tan SB, Fung D, Chia KS, Koh D, Tan DT, et al IQ and the association with myopia in children. Investigative ophthalmology & visual science. 2004;45(9):2943–8. 15326105 10.1167/iovs.03-1296

[47] Morgan IG, Rose KA. Myopia and international educational performance. Ophthalmic & physiological optics: the journal of the British College of Ophthalmic Opticians (Optometrists). 2013;33(3):329–38. 23662964 10.1111/opo.12040

[48] Jung SK, Lee JH, Kakizaki H, Jee D. Prevalence of myopia and its association with body stature and educational level in 19-year-old male conscripts in seoul, South Korea. Investigative ophthalmology & visual science. 2012;53(9):5579–83. 22836765 10.1167/iovs.12-10106

[49] Hashemi H, Nabovati P, Malekifar A, Yekta A, Ostadimoghaddam H, Jafarzadehpur E, et al Astigmatism in underserved rural areas: a population based study. Ophthalmic & physiological optics: the journal of the British College of Ophthalmic Opticians (Optometrists). 2016;36(6):671–9. 27572485 10.1111/opo.12317

[50] Jonas JB, Bi HS, Wu JF, Xu L, Wang YX, Wei WB, et al Corneal Curvature Radius in Myopia of Schoolchildren Versus Adult Myopia. Cornea. 2016;35(10):1333–7. 10.1097/ICO.0000000000000854 27100659

[51] Lim LS, Saw SM, Jeganathan VS, Tay WT, Aung T, Tong L, et al Distribution and determinants of ocular biometric parameters in an Asian population: the Singapore Malay eye study. Investigative ophthalmology & visual science. 2010;51(1):103–9. 19684013 10.1167/iovs.09-3553

[52] Lee KE, Klein BE, Klein R, Quandt Z, Wong TY. Association of age, stature, and education with ocular dimensions in an older white population. Archives of ophthalmology (Chicago, Ill: 1960). 2009;127(1):88–93. 10.1001/archophthalmol.2008.521 PMC2725427 19139346

[53] Verhoeven VJ, Hysi PG, Wojciechowski R, Fan Q, Guggenheim JA, Hohn R, et al Genome-wide meta-analyses of multiancestry cohorts identify multiple new susceptibility loci for refractive error and myopia. Nat Genet. 2013;45(3):314–8. 10.1038/ng.2554 23396134 PMC3740568

[54] Jonas JB, Xu L, Wang YX, Bi HS, Wu JF, Jiang WJ, et al Education-Related Parameters in High Myopia: Adults versus School Children. PloS one. 2016;11(5):e0154554 10.1371/journal.pone.0154554 27152764 PMC4859491

