# Supplementary material for: Myopia is associated with education: Results from NHANES 1999-2008
Source: PLoS One. 2019 Jan 29;14(1):e0211196. doi: 10.1371/journal.pone.0211196 (PMC6350963; doi:10.1371/journal.pone.0211196)
Supplement: S1 Table — (PDF) [file pone.0211196.s001.pdf]

S1 Table. Comparison of the NHANES 1999 – 2008 sample without and with self-reported refractive surgery for near sightedness (excluded from analysis).

	NO REFRACTIVE SURGERY	REFRACTIVE SURGERY
**N**	20,956	374
**AGE**	48.09 (17.57)	48.67 (15.29)
**FEMALE SEX (N, %)**	10830 (51.7)	219 (58.6)
**FAMILY INCOME TO POVERTY INDEX**	2.64 (1.61)	3.64 (1.57)
**EDUCATION:**		
**LESS THAN 9TH GRADE (N, %)**	2718 (13.0)	41 (11.0)
**9-11TH GRADE (N, %)**	3499 (16.7)	32 (8.6)
**HIGH SCHOOL GRAD/GED OR EQUIVALENTA (N, %)**	5101 (24.3)	55 (14.7)
**SOME COLLEGE OR AA DEGREEA (N, %)**	5648 (27.0)	95 (25.4)
**COLLEGE GRADUATE OR ABOVEA (N, %)**	3990 (19.0)	151 (40.4)
**ETHNICITY:**		
**MEXICAN AMERICAN (N, %)**	4460 (21.3)	67 (17.9)
**OTHER HISPANIC (N, %)**	1219 (5.8)	19 (5.1)
**NON-HISPANIC WHITE (N, %)**	10239 (48.9)	231 (61.8)
**NON-HISPANIC BLACK (N, %)**	4236 (20.2)	40 (10.7)
**OTHER (N, %)**	802 (3.8)	17 (4.5)

[a]self-reported; AA: Associate of Arts degree, undergraduate academic degree awarded by colleges usually after completion of a two-year course; GED: General Education Development or Diploma, certification that provides that the test taker has United States or Canadian high-school-level academic skills.
