# Supplementary material for: Myopia is associated with education: Results from NHANES 1999-2008
Source: PLoS One. 2019 Jan 29;14(1):e0211196. doi: 10.1371/journal.pone.0211196 (PMC6350963; doi:10.1371/journal.pone.0211196)
Supplement: S2 Table — (PDF) [file pone.0211196.s002.pdf]

S2 Table. Characteristics of the NHANES 1999 – 2008 sample with age, sex, education and refraction (right eye) available, by ethnicity (n = 19,756).

	Mexican American[a]	Other Hispanic[a]	Non-Hispanic White[a]	Non-Hispanic Black[a]	Other[a]
n	4290	1168	9446	4081	771
Age	44.13 (16.33)	44.47 (15.77)	48.64 (17.41)	45.71 (15.87)	43.01 (15.34)
Female sex	2234 (52.1)	640 (54.8)	4791 (50.7)	2107 (51.6)	406 (52.7)
Family income to poverty index[a]	1.98 (1.41)	2.08 (1.44)	3.10 (1.61)	2.44 (1.57)	2.72 (1.68)
**Education[a]:**					
Less Than 9th Grade[a]	1524 (35.5)	241 (20.6)	403 (4.3)	219 (5.4)	70 (9.1)
9-11th Grade (Includes 12th grade with no diploma) [a]	915 (21.3)	244 (20.9)	1001 (10.6)	1029 (25.2)	86 (11.2)
High School Grad/GED or Equivalent[a]	816 (19.0)	226 (19.3)	2625 (27.8)	982 (24.1)	156 (20.2)
Some College or AA degree[a]	770 (17.9)	298 (25.5)	2865 (30.3)	1253 (30.7)	211 (27.4)
College Graduate or above[a]	265 (6.2)	159 (13.6)	2552 (27.0)	598 (14.7)	248 (32.2)
**Ocular characteristics**					
Sphere [D]	-0.72 (1.98)	-0.68 (2.18)	-1.00 (2.48)	-0.91 (2.27)	-1.29 (2.62)
Cylinder [D]	0.85 (0.91)	0.82 (0.80)	0.85 (0.75)	0.84 (0.74)	0.75 (0.69)
Spherical Equivalent [D]	-0.29 (1.83)	-0.27 (2.07)	-0.57 (2.42)	-0.49 (2.19)	-0.91 (2.56)
Spherical Equivalent <=-0.75D (%)	1112 (25.9)	297 (25.4)	3264 (34.6)	1231 (30.2)	285 (37.0)
Spherical Equivalent (%)					
>-0.75 D	3178 (74.1)	871 (74.6)	6182 (65.4)	2850 (69.8)	486 (63.0)
-0.75/-3 D	836 (19.5)	201 (17.2)	1982 (21.0)	852 (20.9)	156 (20.2)
-3/-6 D	211 (4.9)	70 (6.0)	952 (10.1)	279 (6.8)	77 (10.0)
<=-6 D	65 (1.5)	26 (2.2)	330 (3.5)	100 (2.5)	52 (6.7)
Distance glasses (%)	1422 (33.2)	465 (39.8)	5318 (56.3)	1636 (40.1)	373 (48.4)
Visual acuity [logMAR]	0.15 (0.22)	0.15 (0.22)	0.12 (0.18)	0.14 (0.21)	0.15 (0.23)

[a]self-reported; AA: Associate of Arts degree, undergraduate academic degree awarded by colleges usually after completion of a two-year course; GED: General Education Development or Diploma, certification that provides that the test taker has United States or Canadian high-school-level academic skills.
