# Supplementary material for: Myopia is associated with education: Results from NHANES 1999-2008
Source: PLoS One. 2019 Jan 29;14(1):e0211196. doi: 10.1371/journal.pone.0211196 (PMC6350963; doi:10.1371/journal.pone.0211196)
Supplement: S3 Table — (PDF) [file pone.0211196.s003.pdf]

**S3 Table. The association of spherical equivalent with education in separate models in the NHANES 1999 – 2008, with additional adjustment.**

| | Adjusted model[a] (n=19,756) | | Adjusted model[b1] (n=19,704) | | Adjusted model[b2] (n= 14,651) | |
|---|---|---|---|---|---|---|
| **Education** | **Estimate in diopter [95% confidence interval]** | **P value** | **Estimate in diopter [95% confidence interval]** | **P value** | **Estimate in diopter [95% confidence interval]** | **P value** |
| Less Than 9th Grade | Reference | - | Reference | - | Reference | - |
| 9-11th Grade | -0.23 [-0.37; -0.08] | 0.01 | -0.21 [-0.35; -0.07] | 0.01 | -0.23 [-0.38; -0.08] | 4.56e-03 |
| High School Grad/GED or Equivalent | -0.45 [-0.61; -0.30] | 1.59e-07 | -0.38 [-0.53; -0.22] | 1.36e-05 | -0.48 [-0.65; -0.30] | 2.67e-06 |
| Some College or AA degree | -0.76 [-0.90; -0.61] | 3.36e-15 | -0.65 [-0.79; -0.51] | 6.20e-13 | -0.77 [-0.93; -0.61] | 3.00e-12 |
| College Graduate or above | -1.43 [-1.59; -1.26] | < 2e-16 | -1.24 [-1.40; -1.07] | < 2e-16 | -1.39 [-1.58; -1.19] | < 2e-16 |

All models calculated with consideration of the study sample structure; [a] results from the multivariable linear regression model adjusted for age, sex, survey cycle, corneal power, ethnicity; [b1] additionally adjusted for poverty-to-income ratio; [b2] additionally adjusted for vitamin d level as a proxy for outdoor activity; AA: Associate of Arts degree, undergraduate academic degree awarded by colleges usually after completion of a two-year course; GED: General Education Development or Diploma, certification that provides that the test taker has United States or Canadian high-school-level academic skills.
