# Supplementary material for: Myopia is associated with education: Results from NHANES 1999-2008
Source: PLoS One. 2019 Jan 29;14(1):e0211196. doi: 10.1371/journal.pone.0211196 (PMC6350963; doi:10.1371/journal.pone.0211196)
Supplement: S4 Table — (PDF) [file pone.0211196.s004.pdf]

S4 Table. The association of myopia (≤ -0.75 D) with education in separate models in the NHANES 1999 – 2008, with additional adjustment.

	Adjusted model[a] (n= 19,704)		Adjusted model[b1] (n=18,232)		Adjusted model[b2] (n= 14,651)	
**Education**	**Odds ratio [95% confidence interval]**	**P value**	**Odds ratio [95% confidence interval]**	**P value**	**Odds ratio [95% confidence interval]**	**P value**
Less Than 9th Grade	Reference	-	Reference	-	Reference	-
9-11th Grade	1.26 [1.01; 1.57]	0.05	1.29 [1.02; 1.63]	0.04	1.22 [0.97; 1.54]	0.09
High School Grad/GED or Equivalent	1.63 [1.34; 1.97]	5.46e-06	1.61 [1.32; 1.95]	1.52e-05	1.61 [1.31; 1.97]	4.12e-05
Some College or AA degree	2.23 [1.83; 2.72]	7.19e-11	2.15 [1.75; 2.64]	7.99e-10	2.20 [1.78; 2.73]	4.05e-09
College Graduate or above	3.60 [2.95, 4.40]	< 2e-16	3.27 [2.64; 4.07]	1.65e-15	3.48 [2.81; 4.30]	4.82e-15

All models calculated with consideration of the study sample structure; [a] results from the multivariable linear regression model adjusted for age, sex, survey cycle, corneal power, ethnicity; [b1] additionally adjusted for poverty-to-income ratio; [b2] additionally adjusted for vitamin d level as a proxy for outdoor activity; AA: Associate of Arts degree, undergraduate academic degree awarded by colleges usually after completion of a two-year course; GED: General Education Development or Diploma, certification that provides that the test taker has United States or Canadian high-school-level academic skills.
