# Supplementary material for: Myopia is associated with education: Results from NHANES 1999-2008
Source: PLoS One. 2019 Jan 29;14(1):e0211196. doi: 10.1371/journal.pone.0211196 (PMC6350963; doi:10.1371/journal.pone.0211196)
Supplement: S5 Table — (PDF) [file pone.0211196.s005.pdf]

S5 Table. Sensitivity analysis: The association of spherical equivalent with education in separate models in the NHANES 1999 – 2008, restricted to participants aged 30 years and older

	Crude analysis (n= 15,620)		Adjusted model[a] (n=15,620)		Adjusted model[b] (n=15,578)	
**Education**	**Estimate in diopter [95% confidence interval]**	**P value**	**Estimate in diopter [95% confidence interval]**	**P value**	**Estimate in diopter [95% confidence interval]**	**P value**
Less Than 9th Grade	Reference	-	Reference	-	Reference	-
9-11th Grade	-0.43[-0.58; -0.28]	5.33e-07	-0.22 [-0.35; -0.08]	0.002	-0.24 [-0.39; -0.09]	0.002
High School Grad/GED or Equivalent	-0.72 [-0.88; -0.56]	9.13e-13	-0.54 [0.68; -0.39]	6.82e-10	-0.53 [-0.68; -0.37]	3.63e-09
Some College or AA degree	-1.07 [-1.23; -0.91]	< 2e-16	-0.80 [-0.94; -0.65]	3.12e-16	-0.79 [-0.94; -0.64]	2.00e-15
College Graduate or above	-1.69 [-1.88; -1.50]	< 2e-16	-1.42 [-1.59; -1.24]	< 2e-16	-1.42 [-1.60; -1.24]	< 2e-16

All models calculated with consideration of the study sample structure; [a] results from the multivariable linear regression models adjusted for age, sex, survey cycle; [b] additionally adjusted for corneal power; AA: Associate of Arts degree, undergraduate academic degree awarded by colleges usually after completion of a two-year course; GED: General Education Development or Diploma, certification that provides that the test taker has United States or Canadian high-school-level academic skills.
