# Supplementary material for: Myopia is associated with education: Results from NHANES 1999-2008
Source: PLoS One. 2019 Jan 29;14(1):e0211196. doi: 10.1371/journal.pone.0211196 (PMC6350963; doi:10.1371/journal.pone.0211196)
Supplement: S6 Table — (PDF) [file pone.0211196.s006.pdf]

S6 Table. Sensitivity analysis: The association of myopia (≤ -0.75 D) with education in separate models in the NHANES 1999 – 2008, restricted to participants aged 30 years and older.

	Crude analysis (n= 15,620)		Adjusted model[a] (n=15,620)		Adjusted model[b] (n=15,578)	
**Education**	**Odds ratio [95% confidence interval]**	**P value**	**Odds ratio [95% confidence interval]**	**P value**	**Odds ratio [95% confidence interval]**	**P value**
Less Than 9th Grade	Reference	-	Reference	-	Reference	-
9-11th Grade	1.49 [1.19; 1.87]	0.001	1.36 [1.08; 1.70]	0.01	1.38 [1.09; 1.76]	0.01
High School Grad/GED or Equivalent	2.08 [1.71; 2.54]	3.36e-10	1.92 [1.58; 2.34]	1.35e-08	1.92 [1.56; 2.36]	6.30e-08
Some College or AA degree	2.91 [2.35; 3.61]	9.44e-15	2.56 [2.06; 3.18]	3.59e-12	2.59 [2.06; 3.25]	1.42e-11
College Graduate or above	4.48 [3.62; 5.54]	< 2e-16	4.01 [3.24; 4.96]	< 2e-16	4.13 [3.30; 5.16]	< 2e-16

All models calculated with consideration of the study sample structure; [a] results from the multivariable logistic regression models adjusted for age, sex, survey cycle; [b] additionally adjusted for corneal power; AA: Associate of Arts degree, undergraduate academic degree awarded by colleges usually after completion of a two-year course; GED: General Education Development or Diploma, certification that provides that the test taker has United States or Canadian high-school-level academic skills.
