# Supplementary material for: Myopia is associated with education: Results from NHANES 1999-2008
Source: PLoS One. 2019 Jan 29;14(1):e0211196. doi: 10.1371/journal.pone.0211196 (PMC6350963; doi:10.1371/journal.pone.0211196)
Supplement: S7 Table — (PDF) [file pone.0211196.s007.pdf]

S7 Table. The association of spherical equivalent with education in different ethnicities in the NHANES 1999 – 2008.

	Mexican Americana (n= 4,280)		Other Hispanic[a] (n=1,164)		Non-Hispanic White[a] (n= 9,426)		Non-Hispanic Black[a] (n=4,066)		Other[a] (n=768)	
**Education**	**Estimate in diopter [CI]**	**P value**	**Estimate in diopter [CI]**	**P value**	**Estimate in diopter [CI]**	**P value**	**Estimate in diopter [CI]**	**P value**	**Estimate in diopter [CI]**	**P value**
Less Than 9th Grade	Reference	-	Reference	-	Reference	-	Reference	-	Reference	-
9-11th Grade	-0.18 [-0.32; -0.03]	0.02	-0.24 [-0.60, 0.11]	0.18	-0.24 [-0.50; 0.02]	0.08	-0.28 [-0.59, 0.04]	0.08	0.06 [-0.70, 0.83]	0.87
High School Grad/GED or Equivalent	-0.40 [-0.55; -0.25]	1.88e-07	-0.35 [-0.71, 0.00]	0.05	-0.47 [-0.71; -0.23]	1.14e-04	-0.53 [-0.84; -0.21]	1.05e-03	-0.33 [-1.01, 0.35]	0.34
Some College or AA degree	-0.70 [-0.85; -0.55]	< 2e-16	-0.72 [-1.06, -0.38]	3.00e-05	-0.78 [-1.02; -0.54]	2.21e-10	-0.63 [-0.95, -0.32]	6.7e-05	-0.82 [-1.47, -0.16]	0.02
College Graduate or above	-0.96 [-1.19; -0.74]	< 2e-16	-0.85 [-1.24, -0.46]	2.06e-05	-1.46 [-1.70; -1.21]	< 2e-16	-1.19 [-1.52, -0.86]	2.1e-12	-1.38 [-2.02, -0.74]	2.75e-05

[a] Multivariable linear regression model results adjusted for age, sex, survey cycle, corneal power; CI: 95% confidence interval; AA: Associate of Arts degree, undergraduate academic degree awarded by colleges usually after completion of a two-year course; GED: General Education Development or Diploma, certification that provides that the test taker has United States or Canadian high-school-level academic skills.
