# Supplementary material for: Myopia is associated with education: Results from NHANES 1999-2008
Source: PLoS One. 2019 Jan 29;14(1):e0211196. doi: 10.1371/journal.pone.0211196 (PMC6350963; doi:10.1371/journal.pone.0211196)
Supplement: S8 Table — (PDF) [file pone.0211196.s008.pdf]

S8 Table. The association of myopia (≤ -0.75 D) with education in different ethnicities in the NHANES 1999 – 2008.

	Mexican American (n=4,280)		Other Hispanic (n=1,164)		Non-Hispanic White (n=9,426)		Non-Hispanic Black (n=4,066)		Other (n=768)	
**Education**	**Odds ratio [CI]**	**P value**	**Odds ratio [CI]**	**P value**	**Odds ratio [CI]**	**P value**	**Odds ratio [CI]**	**P value**	**Odds ratio [CI]**	**P value**
Less Than 9th Grade	Reference	-	Reference	-	Reference	-	Reference	-	Reference	-
9-11th Grade	1.22 [0.99; 1.50]	0.06	1.64 [1.01; 2.71]	0.05	1.15 [0.85; 1.57]	0.38	1.87 [1.21; 3.03]	7.33e-037	0.86 [0.39; 1.95]	0.71
High School Grad/GED or Equivalent	1.66 [1.35 2.04]	1.67e-06	1.76 [1.08; 2.92]	0.03	1.52 [1.15; 2.02]	0.003	2.41 [1.55; 3.89]	1.78e-04	1.28 [0.65; 2.66]	0.49
Some College or AA degree	2.15 [1.75 2.64]	2.17e-13	2.59 [1.65; 4.16]	5.06e-05	2.08 [1.59; 2.77]	2.55e-07	2.71 [1.75; 4.37]	1.77e-05	2.11 [1.10; 4.25]	0.03
College Graduate or above	3.02 [2.27 4.01]	2.93e-14	3.25 [1.97; 5.46]	5.53e-06	3.33 [2.53; 4.43]	< 2e-16	4.65 [2.97; 7.57]	1.09e-10	3.00 [1.59; 5.98]	1.03e-03

Multivariable logistic regression model results adjusted for age, sex, survey cycle, corneal power; CI: 95% confidence interval; AA: Associate of Arts degree, undergraduate academic degree awarded by colleges usually after completion of a two-year course; GED: General Education Development or Diploma, certification that provides that the test taker has United States or Canadian high-school-level academic skills.
