# Supplementary material for: Myopia is associated with education: Results from NHANES 1999-2008
Source: PLoS One. 2019 Jan 29;14(1):e0211196. doi: 10.1371/journal.pone.0211196 (PMC6350963; doi:10.1371/journal.pone.0211196)
Supplement: S9 Table — (PDF) [file pone.0211196.s009.pdf]

S9 Table. The association of spherical equivalent with education in different ethnicities in the NHANES 1999 – 2008, restricted to US-born participants.

	Mexican American[a] (n= 1,738)		Other Hispanic[a] (n=359)		Non-Hispanic White[a] (n= 8,953)		Non-Hispanic Black[a] (n= 3,715)		Other[a] (n= 353)	
**Education**	**Estimate in diopter [CI]**	**P value**	**Estimate in diopter [CI]**	**P value**	**Estimate in diopter [CI]**	**P value**	**Estimate in diopter [CI]**	**P value**	**Estimate in diopter [CI]**	**P value**
**Less Than 9th Grade**	Reference	-	Reference	-	Reference	-	Reference	-	Reference	-
**9-11th Grade**	-0.35 [-0.69; -0.02]	0.04	-0.52 [-1.65; 0.61]	0.37	-0.21 [-0.48; 0.07]	0.14	-0.28 [-0.62; 0.06]	0.11	-0.51 [-1.79; 0.77]	0.44
**High School Grad/GED or Equivalent**	-0.70 [-1.03; -0.37]	3.23e-05	-0.87 [-1.99 0.24]	0.12	-0.44 [-0.69; -0.19]	5.53e-04	-0.53 [-0.87; -0.18]	2.64e-03	-0.80 [-1.97; 0.37]	0.18
**Some College or AA degree**	-0.89 [-1.22; -0.57]	7.29e-08	-1.27 [-2.35 -0.19]	0.02	-0.75 [-1.00; -0.50]	3.83e-09	-0.66 [-1.01; -0.32]	1.42e-04	-1.32 [-2.46; -0.18]	0.02
**College Graduate or above**	-1.10 [-1.50; -0.70]	8.09e-08	-1.35 [-2.53 -0.18]	0.02	-1.44 [-1.69; -1.19]	< 2e-16	-1.16 [-1.53; -0.80]	4.82e-10	-2.07 [-3.26; -0.89]	6.67e-04

[a] Multivariable linear regression model results adjusted for age, sex, survey cycle, corneal power; CI: 95% confidence interval; AA: Associate of Arts degree, undergraduate academic degree awarded by colleges usually after completion of a two-year course; GED: General Education Development or Diploma, certification that provides that the test taker has United States or Canadian high-school-level academic skills.
