# Supplementary material for: Myopia is associated with education: Results from NHANES 1999-2008
Source: PLoS One. 2019 Jan 29;14(1):e0211196. doi: 10.1371/journal.pone.0211196 (PMC6350963; doi:10.1371/journal.pone.0211196)
Supplement: S10 Table — (PDF) [file pone.0211196.s010.pdf]

S10 Table. The association of myopia (≤ -0.75 D) with education in different ethnicities in the NHANES 1999 – 2008, restricted to US-born participants.

	Mexican American (n= 1,738)		Other Hispanic (n= 359)		Non-Hispanic White (n= 8,953)		Non-Hispanic Black (n=3,715)		Other (n=353)	
**Education**	**Odds ratio [CI]**	**P value**	**Odds ratio [CI]**	**P value**	**Odds ratio [CI]**	**P value**	**Odds ratio [CI]**	**P value**	**Odds ratio [CI]**	**P value**
Less Than 9th Grade	Reference	-	Reference	-	Reference	-	Reference	-	Reference	-
9-11th Grade	1.43 [0.93; 2.24]	0.11	2.20 [0.70; 8.48]	0.20	1.08 [0.79; 1.49]	0.63	1.93 [1.20; 3.27]	9.42e-03	1.39 [0.34; 7.23]	0.67
High School Grad/GED or Equivalent	1.86 [1.23; 2.88]	4.14e-03	2.50 [0.81; 9.50]	0.14	1.43 [1.07; 1.92]	0.02	2.45 [1.52; 4.14]	4.33e-04	2.37 [0.68; 11.19]	0.21
Some College or AA degree	2.12 [1.41; 3.25]	4.32e-04	3.56 [1.19; 13.23]	0.03	1.97 [1.49; 2.65]	3.59e-06	2.83 [1.77; 4.78]	3.74e-05	3.67 [1.09; 16.86]	0.05
College Graduate or above	2.88 [1.78; 4.69]	1.77e-05	3.98 [1.24; 15.58]	0.03	3.16 [2.38; 4.24]	4.22e-15	4.48 [2.75; 7.65]	8.03e-09	5.20 [1.49; 24.56]	0.02

Multivariable logistic regression model results adjusted for age, sex, survey cycle, corneal power; CI: 95% confidence interval; AA: Associate of Arts degree, undergraduate academic degree awarded by colleges usually after completion of a two-year course; GED: General Education Development or Diploma, certification that provides that the test taker has United States or Canadian high-school-level academic skills.
