# Supplementary material for: Myopia is associated with education: Results from NHANES 1999-2008
Source: PLoS One. 2019 Jan 29;14(1):e0211196. doi: 10.1371/journal.pone.0211196 (PMC6350963; doi:10.1371/journal.pone.0211196)
Supplement: S11 Table — (PDF) [file pone.0211196.s011.pdf]

**S11 Table. Association analysis of refractive astigmatism with level of education in the NHANES 1999 – 2008.**

| | Refractive astigmatism $J_0$ | | | | Refractive astigmatism $J_{45}$ | | | |
|---|---|---|---|---|---|---|---|---|
| | **Crude analysis (n=19,756)** | | **Adjusted model[a] (n=19,756)** | | **Crude analysis (n=19,756)** | | **Adjusted model[a] (n=19,756)** | |
| **Education** | **B [95% CI]** | **P value** | **B [95% CI]** | **P value** | **B [95% CI]** | **P value** | **B [95% CI]** | **P value** |
| Less Than 9th Grade | Reference | - | Reference | - | Reference | - | Reference | - |
| 9-11th Grade | 0.07 [0.03; 0.11] | 0.0003 | 0.02 [-0.01; 0.06] | 0.25 | -0.01 [-0.03; 0.01] | 0.30 | -0.01 [-0.03; 0.01] | 0.30 |
| High School Grad/GED or Equivalent | 0.04 [0.00; 0.07] | 0.04 | 0.00 [-0.04; 0.03] | 0.90 | -0.04 [-0.06; -0.02] | 0.0004 | -0.04 [-0.06; -0.02] | 0.0005 |
| Some College or AA degree | 0.05 [0.02; 0.08] | 0.005 | -0.01 [-0.04; 0.02] | 0.55 | -0.04 [-0.06; -0.02] | 0.0001 | -0.04 [-0.06; -0.02] | 9.33e-05 |
| College Graduate or above | 0.04 [0.00; 0.07] | 0.04 | 0.00 [-0.04; 0.03] | 0.90 | -0.05 [-0.07; -0.03] | 2.26e-06 | -0.05 [-0.07; -0.03] | 2.01e-06 |

All models calculated with consideration of the study sample structure; [a] results from the multivariable linear regression models adjusted for age, sex, survey cycle; AA: Associate of Arts degree, undergraduate academic degree awarded by colleges usually after completion of a two-year course; GED: General Education Development or Diploma, certification that provides that the test taker has United States or Canadian high-school-level academic skills.
