# Supplementary material for: Myopia is associated with education: Results from NHANES 1999-2008
Source: PLoS One. 2019 Jan 29;14(1):e0211196. doi: 10.1371/journal.pone.0211196 (PMC6350963; doi:10.1371/journal.pone.0211196)
Supplement: S12 Table — (PDF) [file pone.0211196.s012.pdf]

S12 Table. Association analysis of corneal astigmatism (J_0 and J_{45} vector) with level of education in the NHANES 1999 – 2008.

	Corneal astigmatism J_0				Corneal astigmatism J_{45}			
	**Crude analysis (n=19,703)**		**Adjusted model[a] (n=19,703)**		**Crude analysis (n=19,703)**		**Adjusted model[a] (n=19,703)**	
**Education**	**B [95% CI]**	**P value**	**B [95% CI]**	**P value**	**B [95% CI]**	**P value**	**B [95% CI]**	**P value**
Less Than 9th Grade	Reference	-	Reference	-	Reference	-	Reference	-
9-11th Grade	0.08 [0.05; 0.12]	6.37 e-06	0.03 [0.00; 0.07]	0.045	-0.01 [-0.04; 0.02]	0.47	-0.02 [-0.05; 0.01]	0.25
High School Grad/GED or Equivalent	0.06 [0.03; 0.09]	0.0004	0.02 [-0.01; 0.05]	0.20	-0.05 [-0.08; -0.02]	0.0006	-0.05 [-0.08; -0.02]	0.0004
Some College or AA degree	0.07 [0.03; 0.10]	0.0001	0.00 [-0.02; 0.03]	0.76	-0.06 [-0.09; -0.03]	1.70 e-05	-0.06 [-0.09; -0.04]	1.08 e-05
College Graduate or above	0.03 [0.00; 0.07]	0.03	-0.01 [-0.03; 0.02]	0.68	-0.07 [-0.09; -0.04]	5.13 e-07	-0.07 [-0.10; -0.05]	3.79 e-07

All models calculated with consideration of the study sample structure; *results from the multivariable linear regression models adjusted for age, sex, survey cycle; AA: Associate of Arts degree, undergraduate academic degree awarded by colleges usually after completion of a two-year course; GED: General Education Development or Diploma, certification that provides that the test taker has United States or Canadian high-school-level academic skills.
